# Acceptability and Willingness to Use the “CaknaStrok” Mobile Health Application and Associated Factors Among Informal Caregivers of Post-Stroke Patients in Malaysia: Protocol for an Explanatory Sequential Mixed-Methods Study

**DOI:** 10.3390/healthcare14172874

**Published:** 2026-09-07

**Authors:** Anwar Fazal Abu Bakar, Azimatun Noor Aizuddin, Roszita Ibrahim, Kamarul Imran Musa, Sureshkumar Kamalakannan

**Affiliations:** 1 Department of Community Health, Faculty of Medicine, Universiti Kebangsaan Malaysia (UKM), Kuala Lumpur 56000, Malaysia; azimatunnoor@hctm.ukm.edu.my (A.N.A.); roszita@hctm.ukm.edu.my (R.I.); 2 Department of Community Medicine, School of Medical Sciences, Universiti Sains Malaysia (USM), Kubang Kerian 16150, Malaysia; drkamarul@usm.my; 3 Department of Social Work, Education and Community Wellbeing, Northumbria University, Newcastle upon Tyne NE7 7XA, UK; sureshkumar.kamalakannan@northumbria.ac.uk

**Keywords:** mHealth, mobile health application, acceptability, willingness to use, digital health literacy, informal caregivers, stroke, mixed methods, PLS-SEM, Malaysia

## Abstract

**Background**: Stroke is the third leading cause of death and a major cause of long-term disability in Malaysia, where the continuity of post-stroke care depends heavily on unpaid, untrained informal family caregivers. These caregivers face a well-documented “discharge cliff,” assuming complex medical and rehabilitative responsibilities with minimal preparation. The locally developed “CaknaStrok” mobile health (mHealth) application was designed to bridge this support gap. However, the existence of a tool does not guarantee its use; sustained adoption depends on whether caregivers find the application acceptable and are willing to integrate it into an already demanding caregiving routine. **Objectives**: This protocol describes a study designed to determine the levels of acceptability and willingness to use the CaknaStrok application, to examine the associated predictors (effort expectancy, ability, digital health literacy, and intervention coherence), and to test the central mediating role of acceptability through a novel Integrated Acceptability–Willingness (IAW) Framework. **Methods**: An explanatory sequential mixed-methods design will be conducted at the Stroke Ward of Hospital Canselor Tuanku Muhriz (HCTM), a World Stroke Organization-certified Advanced Stroke Centre. In Phase I, a target of 200 informal primary caregivers will be recruited through consecutive sampling and surveyed using a validated, bilingual (Malay/English) self-administered questionnaire that operationalises the IAW constructs through three established instruments (UTAUT, the Digital Health Literacy Instrument, and the Theoretical Framework of Acceptability). Following a standardised facilitated onboarding, caregivers use the application ad libitum for four to six weeks before completing the questionnaire. **Expected Results**: The study will quantify caregiver acceptability and willingness to use, identify which factors most strongly drive acceptance, and empirically test whether acceptability mediates the relationship between the predictors and willingness to use. Qualitative themes will contextualise the statistical pathways, surfacing culturally specific mechanisms of adoption that single-method designs typically miss. Ethical approval was obtained from the UKM Research Ethics Committee (JEP-2024-392). **Conclusions**: This study is expected to generate context-sensitive evidence to guide the design of caregiver-facing mHealth interventions and inform future multi-site implementation and policy development efforts within the Malaysian stroke-care pathway.

## 1. Introduction

Stroke remains one of the most significant global health challenges. It is currently the second leading cause of death and the third leading cause of disability-adjusted life years (DALYs) worldwide, reflecting its substantial and persistent impact on global health [1,2]. Although advances in acute stroke management have reduced mortality in many high-income countries, the burden of stroke has increasingly shifted towards low- and middle-income countries, where the number of survivors requiring long-term rehabilitation and caregiving continues to rise [3,4].

In Malaysia, stroke is consistently reported as the third leading cause of death, with an estimated 47,000–50,000 new cases occurring annually. National Global Burden of Disease estimates indicate approximately 443,995 prevalent stroke cases and more than 19,000 stroke-related deaths [5,6]. As Malaysia’s population ages and vascular risk factors become increasingly prevalent, the number of stroke survivors returning home with varying degrees of disability continues to increase, creating greater demand for long-term community-based care [7,8].

Recovery following stroke extends far beyond acute hospital treatment and requires sustained physical rehabilitation, medication management, and psychosocial support. Within Malaysia, where formal rehabilitation services remain concentrated in urban tertiary hospitals and post-discharge rehabilitation services are limited, much of this responsibility falls upon informal caregivers, typically spouses or adult children, who provide unpaid care without formal healthcare training [5,9]. Their caregiving role is strongly influenced by cultural values of filial responsibility, including the Malay concept of mengenang budi and the Islamic principle of birr al-wālidayn [10,11]. Consequently, informal caregivers represent the primary intended users of the CaknaStrok mobile application.

CaknaStrok is a non-commercial, evidence-based mobile health (mHealth) application developed by Universiti Sains Malaysia (USM) specifically to support post-stroke caregiving within the Malaysian healthcare context. Developed collaboratively with healthcare professionals, caregivers, and rehabilitation specialists, the application consists of four major functional components: (i) a Guidance module containing 33 educational videos covering stroke knowledge and caregiving skills; (ii) a mental health module incorporating the Depression, Anxiety, and Stress Scale (DASS-21) to provide individualised feedback and referral recommendations; (iii) a location-based health facility finder; and (iv) supplementary functions including the Barthel Index assessment, emergency contacts, support agency directory, and links to official educational resources. By consolidating reliable and locally relevant information into a single platform, CaknaStrok seeks to standardise home-based stroke care while reducing caregiver uncertainty and burden.

Despite the growing availability of mHealth technologies, limited evidence exists regarding the factors influencing caregiver adoption of such interventions in Malaysia. More importantly, an important theoretical gap remains. Established technology acceptance theories, including the Unified Theory of Acceptance and Use of Technology (UTAUT) [12], the Technology Acceptance Model (TAM) [13], and the Theoretical Framework of Acceptability (TFA) [14], each contribute valuable insights into technology adoption but generally conceptualise acceptability as a static outcome rather than an intervening mechanism. These models also provide a limited explanation of how caregiver capability, including digital health literacy and technical ability, together with intervention understanding (intervention coherence), influences willingness to use digital health technologies, particularly within collectivist and family-oriented caregiving cultures in Southeast Asia [15,16]. Furthermore, previous research has been dominated by quantitative studies that identify statistical associations but provide a limited understanding of the contextual factors underlying caregivers’ technology adoption decisions [17]. An additional challenge in caregiver-facing mHealth interventions is the potential creation of a digital divide [18]. Because participation requires access to a compatible smartphone, basic literacy in Malay or English, and the ability to operate digital applications, caregivers who are older, socioeconomically disadvantaged, or have limited digital skills may be excluded from both the intervention and the study itself. This technology-based eligibility boundary may inadvertently reinforce existing health inequalities, particularly in resource-limited settings where access to digital devices and digital literacy remains uneven. Recognising this limitation is important when interpreting the generalisability of the study findings and considering the future implementation of CaknaStrok beyond well-resourced urban healthcare settings. Accordingly, this study addresses the lack of Malaysia-specific, theory-driven, mixed-methods evidence regarding the determinants influencing caregivers’ acceptability of and willingness to use the CaknaStrok application. Specifically, the study aims to investigate the acceptability and willingness to use the CaknaStrok mobile health application among informal caregivers of post-stroke patients in Malaysia.

The specific objectives are as follows:

1. Determine the level of acceptability and willingness to use the CaknaStrok application among informal post-stroke caregivers.
2. Determine the levels of effort expectancy, technical ability, digital health literacy, and intervention coherence among caregivers.
3. Examine the relationships between effort expectancy, technical ability, digital health literacy, intervention coherence, and acceptability.
4. Examine the relationship between acceptability and willingness to use the application.
5. Evaluate the mediating role of acceptability in the relationships between the predictor variables and willingness to use.
6. Explore the issues, challenges, and facilitators that explain caregivers’ acceptability of and willingness to use the CaknaStrok application.

## 2. Materials and Methods

This study adopts an explanatory sequential mixed-methods design [17], in which quantitative data collection and analysis are conducted first, followed by a qualitative phase designed to explain and elaborate upon the quantitative findings. During Phase I, the Integrated Acceptability–Willingness (IAW) Framework is quantitatively examined to determine the levels of each construct and the structural relationships among them. Subsequently, Phase II explores caregivers’ lived experiences to explain the statistical findings and provide contextual understanding of the observed relationships. This sequential approach enables methodological triangulation and strengthens the validity, credibility, and comprehensiveness of the study findings [17,19].

The study is conducted at the Stroke Ward of Hospital Canselor Tuanku Muhriz (HCTM), a tertiary teaching hospital of Universiti Kebangsaan Malaysia (UKM) in Kuala Lumpur. HCTM is recognised as the only hospital in Malaysia, and one of three institutions in the ASEAN region, certified as an Advanced Stroke Centre by the World Stroke Organization. A deliberate single-site approach is adopted to ensure methodological coherence: the same ward serves both as the recruitment hub for the Phase I cohort during admission and as the sampling source for the Phase II post-discharge interviews, thereby preserving cohort continuity across phases. The study runs over 15 months, from 12 November 2025 to 12 February 2027, encompassing instrument validation, piloting, quantitative data collection and analysis, and the subsequent qualitative interviews and integration.

**Figure 1:**
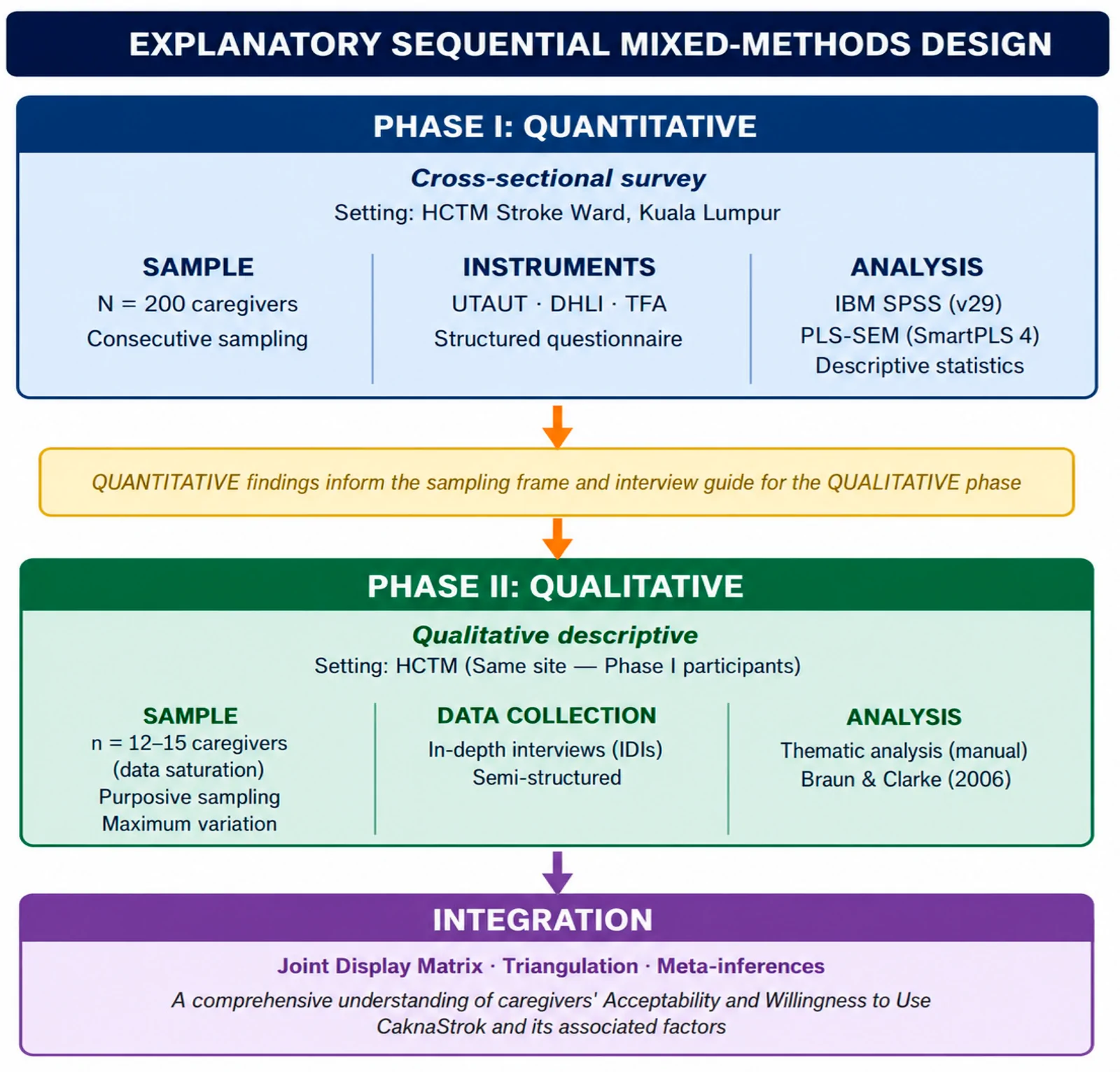
Explanatory sequential mixed-methods study workflow [20].

Figure 1 illustrates the complete explanatory sequential mixed-methods workflow. The study begins with Phase I, the quantitative strand, in which approximately 200 informal caregivers are recruited through consecutive sampling at the HCTM Stroke Ward. Following recruitment and baseline assessment, participants receive standardised onboarding to the CaknaStrok application and use it independently for four to six weeks. They subsequently complete the structured questionnaire measuring effort expectancy, technical ability, digital health literacy, intervention coherence, acceptability, willingness to use, and self-reported app engagement. Quantitative data are analysed using descriptive statistics and PLS-SEM to assess the measurement model, structural relationships, mediation effects, and relevant control variables. The quantitative findings then inform the connection stage between the two phases. Participants are purposively selected from the Phase I cohort using maximum variation in acceptability, digital health literacy, age, caregiver relationship, and app-engagement patterns. Quantitative results also guide the development of qualitative interview probes, particularly for statistically significant, non-significant, or unexpected relationships.

Phase II involves semi-structured in-depth interviews with an initial guideline of approximately 12–15 caregivers. Interviews continue until sufficient information power and data or thematic saturation are achieved. The qualitative data are analysed using reflexive thematic analysis to identify contextual, cultural, and practical explanations for the quantitative findings. Finally, the quantitative and qualitative results are integrated through joint displays and narrative comparison. Areas of convergence, complementarity, and divergence are examined to generate meta-inferences concerning the factors influencing caregivers’ acceptability of and willingness to use CaknaStrok. Thus, the workflow progresses sequentially from quantitative assessment to qualitatively explaining the results and, ultimately, integrating both strands to produce a comprehensive interpretation of caregiver adoption.

### 2.1. Phase I: Quantitative Strand

An a priori power analysis was conducted using G*Power version 3.1.9.7 (F tests; linear multiple regression: fixed model; R2 deviation from zero; a priori). Assuming a significance level of α = 0.05, statistical power of 0.95, a medium effect size of f2 = 0.15, and four predictors, the analysis indicated a minimum required sample of 129 participants [21]. To ensure robustness for partial least squares structural equation modelling (PLS-SEM), the inverse square root method proposed by Kock and Hadaya [22] was also applied. Assuming a minimum path coefficient (|βmin|) of approximately 0.27, the required sample size was estimated using the formula N > (2.486/|βmin|)^2^, yielding a minimum sample requirement of approximately 85 participants. Allowing for approximately 20% attrition during the four- to six-week post-discharge follow-up period and to provide adequate statistical power for mediation analysis and subgroup comparisons, the final target sample size was set at 200 informal caregivers, which substantially exceeds the minimum sample size recommended for robust PLS-SEM analysis [23].

The study instrument consists of a bilingual (Malay/English) self-administered questionnaire comprising demographic information, caregiving characteristics, and measurement scales representing the constructs of the IAW Framework. The questionnaire incorporates validated instruments derived from UTAUT, the Digital Health Literacy Instrument, and the Theoretical Framework of Acceptability [12,14]. Before the main study, all instruments will undergo forward–backward translation, expert content validation, cross-cultural adaptation, and pilot testing to ensure clarity, cultural appropriateness, and measurement validity.

The Phase I instrument is a self-administered, bilingual (Malay/English) structured questionnaire. Sections I–III capture the cohort profile: patient clinical status (modified Rankin Scale [mRS] at discharge, stroke type, and vascular risk factors), caregiver demographics (for stratification), caregiving context (co-residence and use of paid help), and mobile-device readiness (smartphone ownership, operating system, and prior mHealth experience). Sections IV–VI operationalise the IAW constructs through three established instruments, all rated on a 5-point Likert scale (Table 1).

In addition to demographic and caregiving characteristics, the Phase I questionnaire includes three self-reported app-engagement variables within the participant profile and app-usage characteristics section. These variables are included because objective backend usage logs cannot be obtained. Following the four- to six-week exposure period, participants will report the average number of days per week on which they opened or used the CaknaStrok application, the approximate total number of minutes spent using the application on a typical usage day, and the number of weeks for which they continued using the application after onboarding. These items represent app usage frequency, typical usage duration, and continued usage duration, respectively. To illustrate the content of the Phase I questionnaire, representative items include an effort expectancy item assessing whether participants perceive learning to use the CaknaStrok application as easy and a willingness-to-use item assessing whether participants intend to continue using the application. All items are adapted to the CaknaStrok context while retaining the conceptual meaning of the validated instruments from which they were derived. Representative questionnaire items include EE1: “Learning to use the CaknaStrok application is easy for me,” and WU1: “I intend to continue using the CaknaStrok application.” The variables will first be summarised descriptively to describe participants’ level of exposure to CaknaStrok and will subsequently be included as observed control variables with direct paths to willingness to use. This approach will help account for differences in app exposure when estimating the relationships between effort expectancy, technical ability, digital health literacy, intervention coherence, acceptability, and willingness to use.

Immediately following recruitment, participants will receive a standardised onboarding session that includes installation of the CaknaStrok application, user registration, and a structured demonstration of its key functions. Participants will subsequently use the application independently in their home environment for four to six weeks before completing the study questionnaire.

Quantitative data will be analysed using IBM SPSS Statistics version 29 and SmartPLS version 4 [24]. Descriptive statistics will summarise participant characteristics and construct scores. The measurement model will then be assessed for internal consistency reliability, convergent validity, and discriminant validity following current PLS-SEM guidelines [23]. Structural relationships will be evaluated using bootstrapping with 5000 resamples to estimate path coefficients, indirect effects, coefficient of determination (R^2^), effect sizes (f^2^), and predictive relevance (Q^2^). Mediation effects will be interpreted according to the mediation framework proposed by Zhao et al. [25], while common method bias will be assessed using the full collinearity variance inflation factor (VIF) approach [26]. The three self-reported app-engagement measures will be entered as observed control variables with direct paths to willingness to use, thereby controlling for differences in app exposure when estimating the hypothesised structural relationships.

### 2.2. Phase II: Qualitative Strand

The qualitative component (Phase II) adopts purposive maximum-variation sampling to recruit participants from the Phase I cohort. Participants will be selected to ensure diversity in acceptability scores, digital health literacy levels, age, and caregiver relationship to the patient. The qualitative phase will initially aim to recruit approximately 12–15 participants. However, this figure serves only as an initial recruitment guideline and does not represent a fixed sample size. Interviews will continue until sufficient information power is achieved and data or thematic saturation is reached, as indicated by the absence of substantively new information or themes relevant to the research objectives. Therefore, the final sample size may be smaller or larger than the initial estimate, depending on the richness, relevance, and diversity of the data obtained [27,28,29].

1. Inclusion criteria:
  - Completed the Phase I quantitative survey and consented to follow-up contact.
  - Used the CaknaStrok application during the post-discharge period, regardless of frequency or continuity.
  - Willing to participate in a 30–40 min interview.
  - Able to articulate experiences and views in Malay or English.
2. Exclusion criteria:
  - The care recipient (stroke patient) died between Phase I and Phase II.
  - Significant physical or mental health problems precluding interview participation.
  - Withdrawal of consent or refusal to participate in audio-recorded interviews.

The Phase II instrument is a semi-structured in-depth interview guide developed by systematically aligning the quantitative constructs (TFA, DHLI, and UTAUT) with qualitative probes, ensuring theoretical continuity between phases. The guide comprises nine open-ended core questions with associated follow-up probes, organised into six thematic sections: (A) Acceptability and Initial Perceptions (TFA); (B) Ability and Digital Health Literacy (DHLI), including a navigation demonstration via screen-sharing or camera observation; (C) Intervention Coherence (TFA); (D) Burden and Barriers (TFA); (E) Willingness to Use (UTAUT); and (F) Suggestions for Improvement (user experience). All questions are bilingual and tailored to the post-discharge home environment (4–6 weeks post-intervention). Before data collection, a pilot involving three Phase I caregivers assessed the clarity of the guide and the technical feasibility of the remote protocol (audio quality and screen-sharing). Methodological rigour is maintained per the trustworthiness criteria of Guba and Lincoln [30]: credibility through member checking and non-leading probes; dependability through an audit trail documenting all implementation decisions; and confirmability through reflexive memos that record researcher assumptions and potential bias. Qualitative data are collected through semi-structured IDIs conducted via online video-calling platforms (Zoom or Google Meet), scheduled 4–6 weeks after Phase I completion so that responses reflect genuine experience over the usage period rather than immediate post-discharge impressions. Each interview lasts approximately 30–40 min. With participant consent, all sessions are audio-recorded, and field notes capture non-verbal cues; recordings are transcribed verbatim within 72 h, anonymised, and assigned a unique participant code. Semi-structured in-depth interviews will be analysed using reflexive thematic analysis following Braun and Clarke’s six-phase framework [20]. Trustworthiness will be enhanced through member checking, audit trails, reflexive memoing, and confirmability procedures [30].

### 2.3. Mixed-Methods Integration

Integration of quantitative and qualitative findings will occur at both the methods and interpretation stages [17]. Quantitative findings will guide participant selection and interview development for Phase II, while joint displays will be used to integrate statistical findings with qualitative themes to generate meta-inferences regarding caregivers’ acceptability of and willingness to use the CaknaStrok application [18,31]. This integration strengthens methodological triangulation and provides a comprehensive understanding of both the determinants and underlying mechanisms of caregiver technology adoption.

## 3. Conclusions

This protocol presents a theory-driven explanatory sequential mixed-methods study designed to examine the acceptability of and willingness to use the CaknaStrok mHealth application among informal caregivers of post-stroke patients in Malaysia. By positioning acceptability as a mediating mechanism within the Integrated Acceptability–Willingness Framework, the study extends conventional technology-adoption perspectives and provides a more dynamic explanation of how effort expectancy, technical ability, digital health literacy, and intervention coherence may influence caregivers’ willingness to use the application. The quantitative phase will determine the levels of the study constructs, assess their structural relationships, and evaluate the mediating role of acceptability using PLS-SEM. The subsequent qualitative phase will provide deeper insight into the contextual, cultural, and practical factors underlying the quantitative findings. Integrating both phases through joint displays and meta-inferences will enable the study to explain not only which factors influence adoption but also how and why these factors shape caregivers’ experiences and decisions. The study has several methodological strengths, including the use of established theoretical frameworks, standardised application onboarding, real-world post-discharge exposure, cohort continuity across the quantitative and qualitative phases, and systematic mixed-methods integration. Nevertheless, the single-site design, consecutive sampling approach, and reliance on self-reported measures may limit the generalisability of the findings and introduce potential response bias. Because participants are recruited from a highly specialised urban tertiary hospital, the sample may overrepresent caregivers with relatively higher socioeconomic status, healthcare access, and digital readiness. In addition, caregivers who do not own smartphones, have limited Malay or English literacy, or are unable to operate digital devices are unlikely to be represented, creating a technology-based participation boundary that may underestimate barriers faced by more vulnerable populations. However, actual application engagement will not be objectively verified through backend usage logs; acceptability and willingness to use will be assessed primarily through validated self-reported measures.

Despite these limitations, the study is expected to generate contextually relevant evidence for improving the design and implementation of caregiver-focused digital health interventions within the Malaysian stroke-care pathway. The findings may assist application developers in adapting CaknaStrok to caregivers with different levels of digital readiness and caregiving demands. Rather than supporting immediate national-level policy integration, this study aims to build a foundation of evidence for future multi-site implementation studies, particularly in rural and non-urban settings where digital access and healthcare resources may differ substantially. Future implementation efforts should also consider reasonable accommodations for caregivers who are unable to use digital technologies, including printed educational materials, structured telephone follow-up, and ongoing in-person community rehabilitation support.

## Figures and Tables

**Table 1:** Structure of the Phase I questionnaire and IAW construct operationalisation.

Section	Framework	Construct	Item Codes and Number of Items	Response Scale
IV	UTAUT	Effort Expectancy (EE)	EE1–EE5 (5)	5-point Likert (1 = Strongly Disagree to 5 = Strongly Agree)
		Willingness to Use (WU)	WU1–WU4 (4)	
V	DHLI	Digital Health Literacy (DHL; cognitive)	DH1–DH6 (6)	5-point Likert (1 = Very Difficult to 5 = Very Easy)
		Technical Ability (ABI)	DH7–DH10 (4)	
VI	TFA	Intervention Coherence (IC)	IC1–IC4 (4)	5-point Likert (1 = Strongly Disagree to 5 = Strongly Agree)
		Acceptability (ACC)	ACC1–ACC7 (7)	

## Data Availability

The datasets presented in this article are not readily available because the data are part of an ongoing study. Requests to access the datasets should be directed to dr.faizal.a.bakar@gmail.com.

## Abbreviations

The following abbreviations are used in this manuscript:

ABI	Technical Ability
ACC	Acceptability
DALY	Disability-Adjusted Life Year
DHL	Digital Health Literacy
DHLI	Digital Health Literacy Instrument
EE	Effort Expectancy
HCTM	Hospital Canselor Tuanku Muhriz
IAW	Integrated Acceptability–Willingness (Framework)
IC	Intervention Coherence
IDI	In-Depth Interview
mHealth	Mobile Health
mRS	Modified Rankin Scale
PDPA	Personal Data Protection Act
PLS-SEM	Partial Least Squares Structural Equation Modelling
TFA	Theoretical Framework of Acceptability
UKMREC	Universiti Kebangsaan Malaysia Research Ethics Committee
USM	Universiti Sains Malaysia
UTAUT	Unified Theory of Acceptance and Use of Technology
WU	Willingness to Use
